# AFB1 Induced Transcriptional Regulation Related to Apoptosis and Lipid Metabolism in Liver of Chicken

**DOI:** 10.3390/toxins12050290

**Published:** 2020-05-04

**Authors:** Xueqin Liu, Shailendra Kumar Mishra, Tao Wang, Zhongxian Xu, Xiaoling Zhao, Yan Wang, Huadong Yin, Xiaolan Fan, Bo Zeng, Mingyao Yang, Deying Yang, Qingyong Ni, Yan Li, Mingwang Zhang, Qing Zhu, Feng Chen, Diyan Li

**Affiliations:** 1 Institute of Animal Genetics and Breeding, College of Animal Science and Technology, Sichuan Agricultural University, Chengdu 611130, China; liuxueqin@wchscu.cn (X.L.); shailendramphd@gmail.com (S.K.M.); wanttao@hotmail.com (T.W.); zhongxianxu@sicau.edu.cn (Z.X.); zhaoxiaoling@sicau.edu.cn (X.Z.); wangyan519@sicau.edu.cn (Y.W.); yinhuadong@sicau.edu.cn (H.Y.); xiaolanfan@sicau.edu.cn (X.F.); zengbo@sicau.edu.cn (B.Z.); yangmingyao@sicau.edu.cn (M.Y.); Deyingyang@sicau.edu.cn (D.Y.); niqy@sicau.edu.cn (Q.N.); liyan@sicau.edu.cn (Y.L.); 13816@sicau.edu.cn (M.Z.); zhuqing@sicau.edu.cn (Q.Z.); 2 Development Research Center of the State Council, Beijing 100010, China; chenfeng010215@163.com

**Keywords:** aflatoxin B1, Roman layer, liver, high-throughput sequencing, differential expression, fat deposition

## Abstract

Aflatoxin B1 (AFB1) leads to a major risk to poultry and its residues in meat products can also pose serious threat to human health. In this study, after feeding 165-day-old Roman laying hens for 35 days, the toxic effects of aflatoxin B1 at different concentrations were evaluated. The purpose of this study was to explore the mechanism of liver toxicosis responses to AFB1. We found that highly toxic group exposure resulted in liver fat deposition, increased interstitial space, and hepatocyte apoptosis in laying hens. Furthermore, a total of 164 differentially expressed lnRNAs and 186 differentially expressed genes were found to be highly correlated (Pearson Correlation Coefficient > 0.80, *p*-value < 0.05) by sequencing the transcriptome of control (CB) and highly toxic group (TB3) chickens. We also identify 29 differentially expressed genes and 19 miRNAs that have targeted regulatory relationships. Based on the liver cell apoptosis and fatty liver syndrome that this research focused on, we found that the highly toxic AFB1 led to dysregulation of the expression of *PPARG* and *BCL6*. They are cis-regulated by TU10057 and TU45776, respectively. *PPARG* was the target gene of gga-miR-301a-3p, gga-miR-301b-3p, and *BCL6* was the target gene of gga-miR-190a-3p. In summary, highly toxic AFB1 affects the expression levels of protein-coding genes and miRNAs in the liver of Roman layer hens, as well as the expression level of long non-coding RNA in the liver, which upregulates the expression of *PPARG* and downregulates the expression of *Bcl-6*. Our study provides information on possible genetic regulatory networks in AFB1-induced hepatic fat deposition and hepatocyte apoptosis.

## 1. Introduction

Aflatoxin B1 (AFB1) is a secondary fungal metabolite product widely found in many foodstuffs and considered a public health concern worldwide due to its carcinogenic property [1]. There are four primary aflatoxins: aflatoxin B1 (AFB1), B2 (AFB2), G1 (AFG1), and G2 (AFG2). Among them, AFB1 is the most important hepatotoxic, mutagenic and universal food-borne mycotoxin [2]. Aflatoxin production and metabolism, exposure to symptoms and biomarkers, and methods for reducing aflatoxins have been extensively studied [3,4]. Previous studies have been reported that after intake of AFB1, the liver shows obvious fat deposition [5,6]. As studies intensify, other symptoms of AFB1, such as immune damage, oxidative stress, apoptosis, and tumorigenesis, have also been gradually reported [7,8,9].

With the development of technology for RNA sequencing transcripts, we have deepened the understanding of aflatoxin-induced poisoning and provided the goal of regulating the toxicity mechanism. Previous microarray studies have shown that changes in gene expression in chicken livers after exposure to AFB1 diet are associated with energy production and fatty acid metabolism, growth and development, antioxidant protection, coagulation and immune protection, apoptosis and cell proliferation [10,11]. Functional analysis of the turkey hepatic transcription challenged with AFB1 diet showed transcripts dis-regulated by toxicity and affecting pathways of lipid regulation, cell cycle, cancer, and apoptosis [2,12,13,14]. Nowadays, the research on transcription level is not limited to the change in mRNA expression profile, but from RNA-encoding protein to non-coding RNA, such as mRNA and miRNA and lncRNA. Long non-coding RNA (lncRNA) are endogenous of non-coding RNA transcripts > 200 nucleotides (nt) in length that lack an open reading frame [15]. They play crucial role through regulating gene expression in diverse biological processes such as cell differentiation and development, epigenetic regulation, chromatin structure regulation, genomic imprinting, and disease development in mammals [16,17,18,19]. Comparing various non-coding RNAs, e.g., microRNAs, small interfering RNAs etc., lncRNAs are found less conserved than protein coding genes [20]. In poultry, lncRNAs have been identified as having an extensive role in progression of diverse organs and tissue types [21]. The function of lncRNA-MD1 has previously been reported in myogenesis by titration mir-133 and mir-135 [22]. Lnc-DHCR24 was demonstrated as an important regulator of lipid metabolism by interaction with the DHCR24 gene encoding a key enzyme in cholesterol biosynthesis [23]. The expression profile of lncRNAs and mRNAs was generated from Beijing-you cocks and speculated that lncRNA-MSTRG.3652 and MSTRG.4081 were associated with sperm motility in roosters [24]. Long, noncoding RNAs (lncRNAs) also play a key regulatory role in cancer biology. An RNA-seq study on rat liver in response to AFB1 exposure showed that a large group of lncRNAs was highly expressed in the AFB1 hepatocellular carcinogenesis (HCC) by up-regulating anti-apoptosis genes [25]. Other studies showed that the overexpression of certain lncRNAs promote cancer cell matrix invasion and tumor growth [26]. This study indicated that lncRNAs may become a diagnostic marker and therapeutic target for cancer. miRNAs are 19-24 nucleotide (nt) regulatory small RNAs that generally modulate gene expression through translational repression or by causing the degeneration and degradation of target mRNAs to function in many aspects of biological function [27]. Aflatoxin B1 (AFB1) is a mycotoxin that causes liver cancer. It has been observed that some abnormally expressed miRNAs, such as rno-miR-17-92 cluster and rno-miR-34a, are associated with cancer [28]. The overexpression of miR-24 promotes tumor cell proliferation, inhibits apoptosis, and forms AFB1-DNA adducts in hepatocellular carcinoma (HCC) related to aflatoxin B1 (AFB1) [29]. Aflatoxins produce gross and histopathological lesions in different organs, especially liver, kidneys, bursa of Fabricius and muscles [30]. The main functions of the liver in the metabolism include the breakdown of red blood cells, the regulation of glycogen storage, and the production of hormones [31]. In addition, the liver is involved in protein synthesis, the production of biochemical products necessary for digestion, and is responsible for detoxification and immunological functions [32,33]. The liver also has well-defined roles in the maintenance of nutrient homeostasis, the metabolism of xenobiotics and endogenous hormones, and the detoxification of exogenous compounds [34]. Although some of the effects of AFB1 on poultry have been reported by transcription data [11,35] which were mainly focused on mRNA, the regulatory mechanism of lncRNAs, miRNAs and their co-expression with mRNA in response to AFB1 in chicken liver remains unexplored.

As our principal source of protein, chicken is one of the world’s largest meat sources and one of the most widely consumed. Since the introduction of Roman laying hens in China, this breed has not only become the main breed of brown-shell laying hens, it is also the principal source breed for laying hen research. In chicken, AFB1 exposure showed that genes associated with cell proliferation were up-regulated in liver [11]. Considering the liver as the principal target organ of AFB1 and its strong carcinogenicity [36], and progressively more experimental evidence suggests a link between lncRNA/microRNA and mRNA [37,38], this study was designed to determine the change in the chicken liver transcriptome response to the toxic effects of AFB1. We generated differential expression profiles of lncRNA, miRNA and mRNA in the liver tissue of adult Roman chickens with and without AFB1 exposure. The discovered potential candidate genes will not only help to better understand the molecular mechanisms involved in the development of AFB1-induced hepatotoxicity, but also shed new light on diagnosis, as well as enrich the valuable resources for studying chicken lncRNA and miRNA.

## 2. Results

### 2.1. Histopathological Observations of Liver

**Figure 1:**
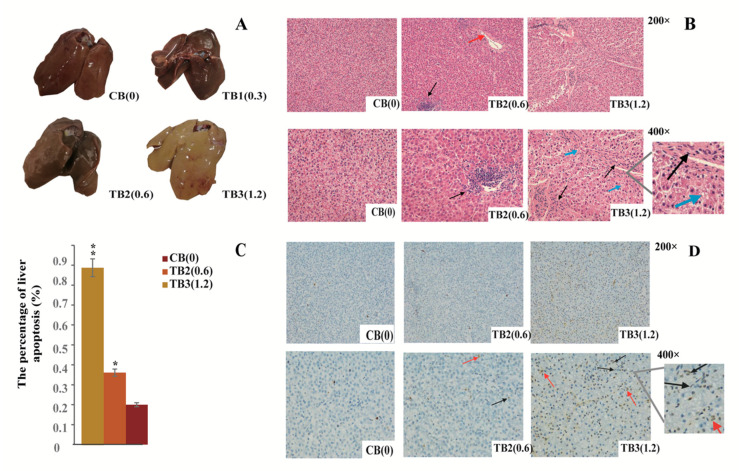
**Liver tissue lesion observation and hepatocyte apoptosis detected by TUNEL assay.** (**a**) liver lesions at different concentrations of AFB1 (mg/kg). (**b**) observation of liver histopathological changes. The red arrow refers to a small amount of liver cells visible in the vein, the black arrow points to the inflammatory cells, and the blue arrow points to the lipid droplets. (**c**) The percentages of apoptosis cells detected by TUNEL (%). Bars represent the standard error. A single asterisk (*p* < 0.05) or two asterisks (*p* < 0.01) above a bar represents a statistically significant difference between the CB and TB2 or TB3. The statistical analyses use one-way ANOVA. (**d**) TUNEL-positive hepatocytes are shown (200×, 400×). The black arrows indicate round, crescent-shaped or irregularly shaped apoptosis cells; the red arrows indicate apoptosis cells with the nucleus condensed in brown.

To investigate the toxicology of AFB1, the liver was routinely stained with haematoxylin-eosin (HE) staining, and histological changes were observed and recorded under a light microscope. Compared to the control group, liver tissues from AFB1-treated chicken appeared swollen with a yellow color appearance (Figure 1a). The toxicity of 0.3mg/kg (TB1) AFB1 on the liver was not obvious, therefore, TB1 excluded from histopathological examination experiment. In other groups, HE staining showed that with the increase of AFB1 concentration, liver hepatocytes were mildly edematous, loose cytoplasm and become pale-stained. There were few hepatocytes in the vein and focal infiltration of inflammatory cells in the TB2 group. In addition, for TB3 group, the hepatocyte gap was significantly enlarged, and the liver was accompanied by lipid droplet formation (Figure 1b).

### 2.2. AFB1 Triggered Apoptosis of Liver

The percentage of apoptosis hepatocytes increased significantly in a dose-dependent manner (Figure 1c). Apoptotic cells in brown, round, crescent or irregular shape were also detected by the terminal deoxynucleotidyl transferase-mediated DUTP nick end labeling (TUNEL) (Figure 1d). AFB1 treatment showed both morphological and molecular level alterations in chicken liver, indicating that AFB1 inhibited hepatocyte growth and the chicken treated with AFB1 had hepatic injury.

### 2.3. Sequencing Data Statistics

We performed lncRNA sequencing on the liver control group (CB) and the treatment group (TB3). A total of 95.01 Gb raw lncRNA data were obtained with an average of 15.84 Gb data measured per sample (Table 1). After stringent filtering the raw sequences, a total of 93.57 Gb clean lncRNA data was obtained with an average of 15.60 Gb of clean data obtained per sample. For miRNA sequencing data, we received a total of 3.46 Gb of raw miRNA data in the liver, with an average of 580 Mb per sample (Table 1). By removing low-quality reads, a total of 2.14 Gb clean data was retained with an average of 357 Mb data per sample ranged from 17 to 36 nt in length.

### 2.4. Sample Correlation Analysis

**Figure 2:**
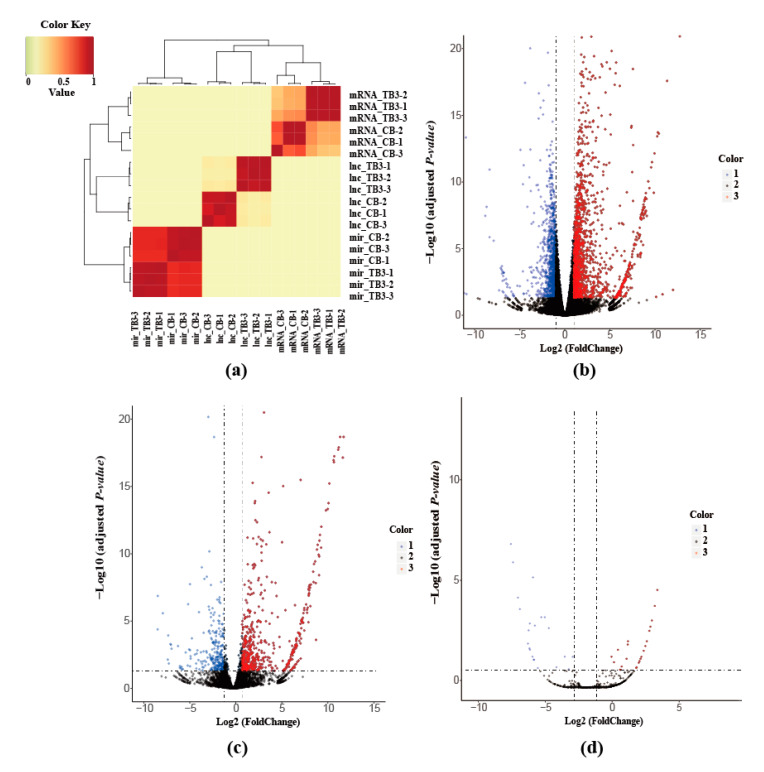
(**a**) Correlation analysis of the expression profile of liver lncRNA miRNA mRNA. (**b**) Differentially expressed mRNAs between CB and TB3. (**c**) Differentially expressed lncRNAs between CB and TB3. (**d**) Differentially expressed miRNAs between CB and TB3. Note: 1 (blue) represents down-regulated genes, 2 (black) represents non-differentiated genes, and 3 (red) represents up-regulated genes.

In total, we found that 6568 lncRNAs, 19,846 mRNAs and 1175 miRNAs were expressed in the chicken liver (Table S1). We then calculated the correlation coefficients based on protein-coding gene, miRNA and lncRNA expression profiles, the correlation coefficients of the control group and the treatment group varied significantly in different types of sequencing data (Figure 2a).

### 2.5. Analysis of Differentially Expressed mRNAs lncRNAs and miRNAs

We next identified and characterized differentially expressed genes (DEGs), lncRNAs and miRNAs in AFB1-treated group compared to the control group (Table S2). The volcano plots revealed variable expression patterns of the differentially expressed genes, lncRNAs and miRNAs. We identified a total of 1927 DEGs consist of 1116 upregulated and 811 downregulated genes (Figure 2b). A total of 867 differentially expressed lncRNAs consist of 570 upregulated and 297 downregulated lncRNAs (Figure 2c) and the 45 differentially expressed miRNAs consist of 23 up regulated and 22 down regulated miRNAs (Figure 2d).

### 2.6. Genomic Characterization of lncRNAs miRNAs and mRNAs

**Figure 3:**
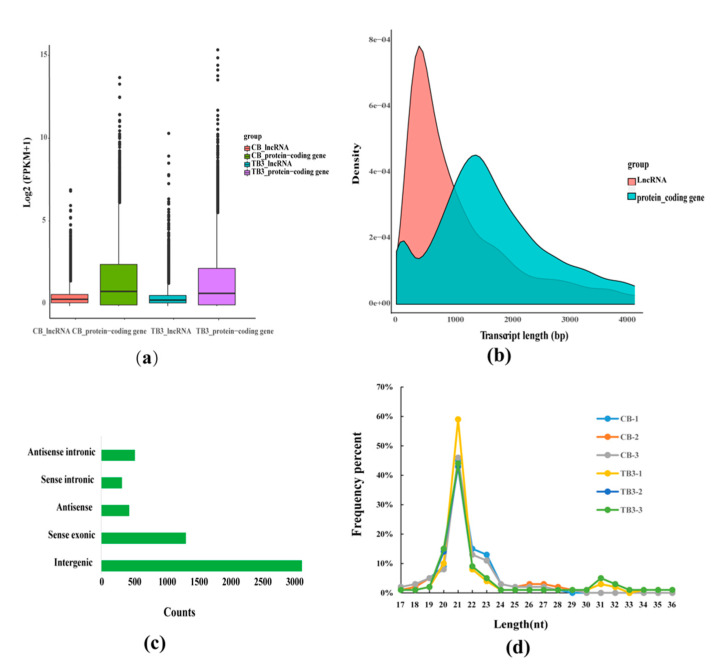
(**a**) Comparison of the expression levels of lncRNAs and mRNAs. (**b**) Distribution of transcript length for lncRNAs and protein-coding genes. (**c**) Classification of lncRNAs. (**d**) Length distribution of miRNA sequences.

We found that lncRNAs were shorter in length and lower in expression than the protein-coding genes (Figure 3a,b). According to their genomic location, the identified lncRNAs were classified by FEELnc software. As a result, 3151 intergenic lncRNAs, 1,329 sense exonic lncRNAs, 439 antisense exonic lncRNAs, 326 sense intronic and 528 antisense intronic lncRNAs were detected (Figure 3c). In general, lncRNA transcripts exhibit low expression levels but tissue-specific expression and are poorly conserved [39]. It is estimated that lncRNAs generally has lower expression levels compared to protein-coding genes [40]. Further, we calculated the base length of the clean data of the miRNA and found that the miRNA length distribution trends of all samples were basically the same, mainly concentrated between 21 to 24 nt. Among them, miRNAs sequences with 21 nucleotides were the most abundant (Figure 3d).

### 2.7. miRNA-mRNA targeted Association Analysis

Based on the results of miRNA and mRNA sequencing data analysis, target genes of differentially expressed miRNAs were predicted by TargetScan and the miRBase database. The predicted target genes which are also DEGs (with |logFC |> 1) were retained, these miRNAs and target genes with high credibility were used for subsequent analysis.

**Figure 4:**
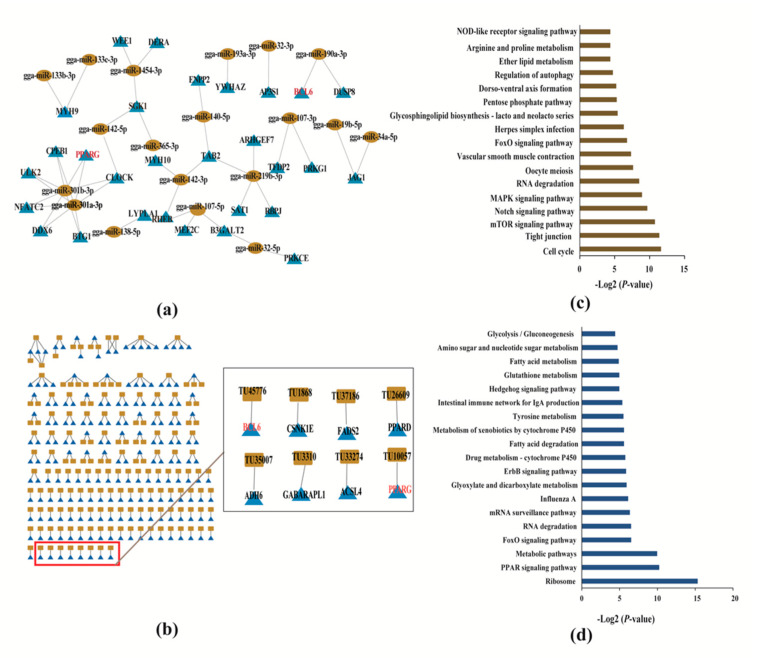
(**a**) Interaction of 47 miRNA–mRNA pairs. Ellipse represent lncRNA, and triangle represent gene. (**b**) Interaction of 216 lncRNA–mRNA pairs. The relationship between lncRNAs-mRNAs interactions is shown in the left, the rectangle represents lncRNA, and triangle represents the gene. (**c**) Functional enrichment pathway of 29 mRNAs regulated by miRNA. (**d**) Functional enrichment pathway of 186 mRNAs regulated by lncRNA.

In total, we identified a total of 29 mRNAs and 19 corresponding miRNAs (Figure 4a), of which 13 upregulated miRNAs corresponded to 20 downregulated targeting mRNAs, and two downregulated miRNAs corresponded to three upregulated mRNAs. The rests are positively regulated, and the targeted correspondence between mRNA and miRNA is shown in Table 2.

### 2.8. Co-expression Network of mRNAs and lncRNAs

The relationship between the DEGs and differentially expressed lncRNAs was further analyzed by Co-expression network. In this study, we screened differentially expressed genes in the vicinity of 867 differentially expressed lncRNAs (upstream and downstream 100kb) as their target genes. Finally, a total of 295 lnRNAs and 369 mRNAs were found to have cis-regulatory relationships. On the other hand, pearson correlation coefficients (PCCs) were calculated to explore the correlations between the expression levels of 295 lncRNAs and 369 mRNAs, PCC > 0.80 and *p* < 0.05 were considered meaningful lncRNA-mRNA pair. Finally, 186 mRNAs and 216 lncRNA-mRNA pairs were found from the co-expression network analysis (Table S3). The co-expression network suggested the inter-regulation among lncRNAs and mRNAs, and all regulating lncRNA–mRNA pairs (Figure 4b). The Pearson correlation coefficient (PCC) and positional information of lncRNA and mRNA are shown in Table S5.

### 2.9. Functional Enrichment Analysis of Target Genes of miRNA and lncRNA

Twenty-nine miRNA target genes were selected for KEGG signaling using KOBAS3.0. We found that the signaling pathways, mTOR signaling pathway and FoxO signaling pathway were significantly enriched (Figure 4c and Table S4). These signaling pathways may lead to the occurrence of apoptosis. DEGs involved in this pathway including *ULK2*, *RHEB*, *SGK1*, and *BCL6* were the major functional genes. Gga-miR-301b-3p, gga-miR-301a-3p, gga-miR-142-3p, gga-miR-365-3p and gga-miR-190a-3p may target these four genes, and plays an important role in the process of apoptosis (Table 2). Further, KEGG analysis of these 189 mRNAs regulated by lncRNA was also performed using KOBAS3.0. Pathways related to fat metabolism such as “PPAR signaling pathway”, “fatty acid degradation” and “fatty acid metabolism” were significantly enriched (Figure 4d and Table S5), of which *ADH6*, *PPARG*, *ACSL4*, *PPARD*, and *FADS2* are major functional genes. In addition, we found that the FoxO signaling pathway was significantly enriched which is associated with apoptosis metabolism. DEGs involved in this pathway including *GABARAPL1* (*ATG8*), *BCL6* and *CSNK1E* were the main functional genes. Finally, we showed the candidate high correlation coefficient lncRNAs-mRNA pairs in Table 3. The cis-regulated lncRNAs associated with these genes may have a regulatory role in AFB1-induced fatty deposition and apoptosis.

### 2.10. Confirmation of RNA-seq results by qRT-PCR

**Figure 5:**
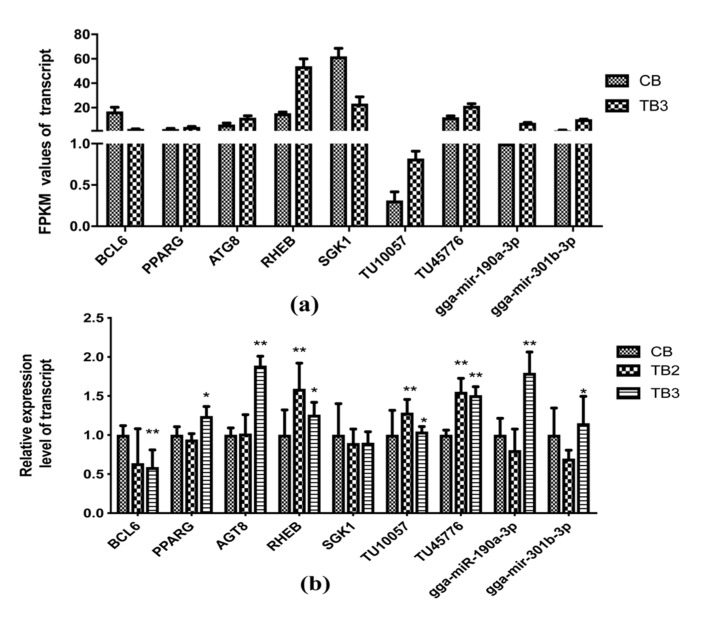
qRT-PCR validated high-throughput sequencing of apoptosis and fat deposition genes. Note: A single asterisk (*p* < 0.05) or two asterisks (*p* < 0.01) represents a statistically significant difference of Reads Per Kilobase per Million mapped reads (FPKM) values between the CB and the TB2 or TB3 chickens. The statistical analyses use one-way ANOVA.

There is a possibility of error in sequencing, and it is necessary to perform qRT-PCR verification again on the expression levels of protein-coding genes of highly related miRNAs and lncRNAs. Based on visual observation of tissue lesions and TUNEL apoptosis detection, we randomly selected some candidate transcripts for qRT-PCR verification and found that the sequencing results were consistent with the expression levels of the transcripts, indicating that our sequencing results are reliable (Figure 5). Aflatoxin at a concentration of 1.2 mg/kg affected key signaling pathways such as the PPAR signaling pathway, fatty acid degradation, fatty acid metabolism, FoxO signaling pathway, etc. in the liver of Roman layer hens. The protein-coding genes involved in these pathways will be targeted and regulated by the non-coding genes lncRNA and miRNA, leading to the up-regulation and down-regulation of these genes to varying degrees. Interestingly, we found that *PPARG* was targeted and regulated simultaneously by one lncRNA (TU10057) and two miRNAs (gga-miR-301a-3p, gga-miR-301b-3p). *BCL6* gene was targeted by lncRNA TU45776 and gga-miR-190a-3p. The results were confirmed by both sequencing and qRT-PCR validation experiments.

## 3. Discussion

Poultry is extremely sensitive to the immune toxic effects of aflatoxin B1 (AFB1). Exposure destroys humoral immunity and limits vaccine efficacy and increases the incidence of secondary infections. The liver is an important detoxifying organ for AFB1 in animals. In our study, after feeding AFB1 feed to hens, the liver with hyperemia and fatty liver was observed with increasing AFB1 concentration. This is consistent with previous reports on the effect of AFB1 on the liver [5,41]. AFB1 was found to able to evoke mitochondrial ROS generation and decreased mitochondrial membrane potential (MMP), induce apoptosis, and increase the percentage of apoptosis cells and expression of caspase-9 and caspase-3 in young chicken [42]. In our study, TUNEL results also showed that a high dose of AFB1 significantly induced liver cell apoptosis. 

In order to better understand the toxicological mechanism of AFB1-induced liver poisoning, we performed high-throughput sequencing of lncRNA and miRNA in the liver of CB (control) and TB3 (high-toxic) groups. We searched for differentially expressed genes within 100 kb upstream and downstream of differentially expressed lncRNAs. Moreover, growing evidence suggests that cis-regulation of lncRNA was investigated earlier than trans-regulation; for instance, H19, AIR, KCNQ1OT1, and XIST were initially discovered lncRNAs [43,44,45,46]. The cis-regulation of lncRNA can regulate the transcription of neighboring genes by facilitating the acquisition of enhancers and promoters of transcriptional machinery molecules, and contribute to the local control of gene expression [47]. Two recently discovered lncRNAs, HOTTIP and HOTAIRM1, are located at both ends of the HoxA cluster and promote adjacent HoxA gene expression by cis-regulation [48,49]. In this study, we found 164 differentially expressed lncRNAs in the vicinity of 186 differentially expressed protein-coding genes, which are likely to alter the expression of these genes by cis-regulation. Further research can prove whether these lncRNAs are actually bound to the promoter region of the target gene in order to achieve regulation of mRNA expression. On the other hand, we also found 29 differentially expressed mRNAs and 19 miRNAs. Not only did they differ significantly in comparison between control and highly AFB1-toxic chickens, the miRNA target genes of were also predicted from the database. This allowed us to screen for more accurate miRNA-regulated protein-coding genes. Although mRNA expression profiles have been studied to determine the effect of AFB1 on chicken liver [11,14], no transcriptome profiles of lncRNAs and miRNAs have been reported to date. All the lncRNAs and miRNAs identified in this study provide further evidence of liver toxicosis responses to AFB1.

We found that many studies have been used for chicks to study the effect of AFB1, which poisoned them with different doses of AFB1. It was found that the higher the concentration of AFB1, the worse the animal’s mental state and the greater the liver damage [50,51]. Early studies have shown that AFB1 is converted to reactive and electrophilic AFB1-8.9-epoxide (AFBO) by the hepatic cytochrome P450 (CYP) enzyme, thereby reducing the toxicity of AFB1 [52,53]. When the liver exceeds its own capacity for detoxification, the animal’s body and internal organs will be severely affected. In this study, we used adult chickens to explore the effect of AFB1 on liver. Based on the functional annotation results of the selected protein-coding genes, we also found that some genes are involved in the signaling pathway related to the cytochrome P450 oxidase system, further confirming the concentration of 1.2 mg/kg AFB1 also causes liver toxicity in laying hens. At the same time, we observed liver cell apoptosis, inflammation, mild fatty liver and so on under the condition of lower concentration (0.6mg/kg) AFB1. Long-term low-dose intake of AFB1 has a harmful effect on the liver [54], so, no matter whether it humans or other animals, it is necessary to avoid AFB1 intake.

In addition, we found pathways related to apoptosis, such as mTOR signaling pathway and FoxO signaling pathway. Previous studies have reported that these signaling pathways are closely related to the occurrence of apoptosis [55,56]. KEGG enrichment pathway analysis of 186 protein-coding genes regulated by lncRNA detected that three pathways, including the PPAR signaling pathway, fatty acid degradation and fatty acid metabolism, were significantly enriched, which are associated with fat metabolism. Lipid metabolism is controlled by multiple pathways and is affected by a variety of genes. In the present study, we found that peroxisome proliferator-activated receptor gamma (*PPARG*) is simultaneously regulated by one lncRNA (TU10057) and two miRNAs (gga-mir-301a-3p, gga-mir-301b-3p). *PPARγ* (also known as *PPARG*) is a member of the peroxisome proliferator-activated receptor (PPAR) subfamily, which was found to have a high expression level in both white and brown fat [57]. The genes activated by *PPARG* are involved in stimulating lipid uptake and fat formation through fat cells. *PPARG* knockout mice do not produce adipose tissue when fed a high-fat diet [58]. Quantitative detection of the expression level of *PPARG* by qRT-PCR showed that the gene was up-regulated and consistent with its sequencing results. Therefore, this interaction network may have important research implications for explaining that AFB1 leads to fatty deposition in the liver. For apoptosis, *BCL6* was simultaneously regulated by both lncRNA (TU45776) and gga-mir-190a-3p, and validation of qRT-PCR was performed for these transcripts. The quantitative expression results of *BCL6*, TU45776 and gga-mir-190a-3p were consistent with the sequencing results. *BCL6* is a diffuse large B-cell lymphatic (DLBCL)-specific marker that functions to prevent cell differentiation and death and promote cell development and proliferation [59]. In our study, we also found that *RHEB* was significantly up-regulated, and *SGK1* and *BCL6* genes were significantly down-regulated. Many reports have confirmed that AFB1 can be induced by apoptosis with increased expression of *bax*, *caspase-3* and *bcl-2* during hepatocellular carcinoma formation [60]. Moreover, we found that AFB1 may also activate the mTORC1 signaling pathway through *RHEB* to induce downstream *SGK1* inactivation and activate FOXO signaling pathway to induce apoptosis-related gene *Bcl-6* downregulation.

## 4. Conclusions

AFB1 exposure induces fat accumulation in liver and apoptosis via regulation of lncRNAs, miRNAs and protein-coding genes. Our results showed that AFB1 mainly induced liver cell apoptosis through the mTOR signaling pathway and FoxO signaling pathway. These signaling pathways were mainly affected by the dysregulation of *ULK2*, *RHEB*, *SGK1*, and *BCL6* expression levels. Among them, co-expression of lncRNA (TU45776), gga-miR-190a-3p and *Bcl-6* gene caused liver cell apoptosis. Fat deposition in the liver is mainly caused by signal pathways such as the “PPAR signaling pathway”, “fatty acid degradation” and “fatty acid metabolism”. These signaling pathways was mainly affected by dysregulation of *ADH6*, *PPARG*, *ACSL4*, *PPARD*, and *FADS2* expression levels. Among them, the co-expression of lncRNA (TU10057), gga-miR-301a-3p, gga-miR-301b-3p and *PPARG* gene caused fatty liver. In summary, the results of this study identified genes and pathways that are differentially altered and provide the first lncRNA and miRNA catalog for aflatoxins in chicken liver.

## 5. Materials and Methods

### 5.1. Experimental Birds and Diets

Chickens were managed according to the Institutional Animal Care and Use Committee of Sichuan Agricultural University under permit number DKY- S20160906, and the approval date was April 18, 2017. In this study, 12 adult Roman hens at 165 days of age from a poultry breeding center (Leshan City, Sichuan Province, China) were used. These birds were divided into four groups, designated as control (CB) and TB1/TB2/TB3 (AFB1-treated), respectively. The chickens in the four groups were supplemented with 0 mg/kg (control, CB), 0.3mg/kg (TB1), 0.6 mg/kg (TB2), and 1.2 mg/kg (TB3) AFB1 for up to 35 days. AFB1 (Sangon, Shanghai, China) was dissolved in DMSO. Chickens in the control group were treated with an equal amount of DMSO to serve as a negative control. All Roman chickens were kept in cages and water was provided ad libitum. Nutritional requirements were adequate according to the 1994 National Research Council guidelines and Chinese chicken feeding standards (NY/T33-2004).

### 5.2. Liver Histopathology

At the end of the experiment, chickens were sacrificed to collect fresh liver tissue less than 1cm thick which was fixed in 4% paraformaldehyde for 48 h. Tissue preparation was performed by alcohol dehydration and embedding in paraffin. After the wax block was fixed in the microtome, the 5-8 μm thick slices were connected to the slides and were dried in a thermostat at 45 °C. The slides were dewaxed with xylene and then hydrated with a mixture of absolute ethanol and xylene (1: 1) and alcohol with a concentration from high to low. The hydrated sections were stained in hematoxylin aqueous solution for 10 min, the cell nuclei were stained blue-violet, and then stained with eosin staining solution for 3 min. Finally, the slices were observed under an optical microscope.

### 5.3. TUNEL Assay

DNA fragmentation indicates that apoptosis signaling cascades were examined using terminal deoxynucleotidyl transferase-mediated DUTP nick end labeling method (TUNEL). The TUNEL assay was performed according to the method described by Tayman et al. [61] and following the manufacturer’s instructions on the TUNEL kit (Roche Molecular Biochemicals, Germany).

### 5.4. RNA Isolation

Total RNA was isolated using TRIzol (Takara Bio, Beijing, China) according to the manufacturer’s protocol. In short, total RNA was isolated from liver tissue (60mg) using 1mL TRIzol (Takara) to lyse the cells. Next, we took the supernatant, added 0.3mL of chloroform for separation, and centrifuged at 13,800× *g* for 10 min at 4 °C. After transferring the upper aqueous phase to another EP tube, 400 μL of pre-chilled isopropanol was added to mix and centrifuge. The supernatant in the tube was discarded, and 1 mL of 75% ethanol pre-cooled at −20 °C was added to wash three times. Finally, 50 μL of Rnase-free water was added to dissolve the RNA and stored in a refrigerator at −80 °C. The quality of total RNA was assessed with Nanodrop ND-1000 spectrophotometer (Bio-Rad, USA) and integrity was further examined using 1% agarose gel electrophoresis.

### 5.5. Illumina Deep Sequencing

To further investigate the changes in liver gene expression levels at high concentrations of AFB1, chickens fed with 0 mg/kg (control) and 1.2 mg/kg (TB3) AFB1 were sequenced. The construction and sequencing of the small RNA library in this study were completed by Haplox (Nanjing, China). Firstly, a small RNA of 17-30 nt in length was collected by gel electrophoresis, followed by a linker sequence at the 5’ and 3’ ends of the small RNA, and a DNA fragment of 70–80 nt in length was obtained by RT-PCR, and then passed through a gel electrophoresis. The product was recovered by electrophoresis and purified. The sequencing was finally performed using the Illumina Novaseq 6000 sequencing platform (Illumina, San Diego, CA, USA). 

The lncRNA library construction in this study was completed by Novogene (Tianjing, China). A total amount of 3 μg RNA per sample was used as the initial material for the RNA sample preparations. Genomic DNA was removed using DNaseI. Six strand-specific cDNA libraries for paired-end 150 bp sequencing were prepared using the dUTP protocol. The library was sequenced on Illumina’s HiSeq platform.

### 5.6. Transcriptomic Data Analysis

The miRNA and miRNA precursor sequences of all species were downloaded from miRBase22; mature miRNA sequences and precursor sequences of the chickens were extracted for subsequent analysis. Sequences of miRNAs from other species were used for the prediction of new miRNAs. The chicken genome (Gallus-gallus-6.0) was also downloaded at ENSEMBL. The chicken genome was indexed using the bowtie software [62], and the miRNA sequencing data were predicted for known miRNAs and new miRNAs using the software miRdeep2 [63]. Counts for each miRNA were calculated by Reads Per Kilobase per Million mapped reads (FPKM), and FPKM > 0.1 was used to filter miRNA.

The filtered high-quality lncRNA reads were mapped to Gallus gallus reference genome using Hisat (v2.0.1) software [64]. The individual transcript was assembled to >200 nucleotides using Stringtie (v1.0.1) software [65]. We combined the transcripts from each biological replicate assembly according to the method described by Iyer et al. (2015) [66]. The transcripts annotated in the reference as “c” and “=” (“c”, partial match and “=”, full match) were excluded by using Cuffcompare 2.2.1 (v2.2.1) [67]. The amino acid sequence was aligned with the Pfam protein family database [68] using the hmmscan software [69], and transcripts with known protein-coding domains were removed. The filtered transcripts were aligned with known protein sequences in existing databases by BLASTX and transcripts with protein coding domains were removed. The Coding Potential Calculator (CPC) [70] predicted the transcript coding potential and removed the transcript with score > 0. Finally, the remaining transcripts with FPKM > 0.1 at least in one biological replicate were annotated as lncRNAs.

### 5.7. Classification of lncRNAs

LncRNAs were classified based on their genomic characterization by FEELnc [71]. The resulting set of lncRNAs was subdivided into five categories: (1) no overlap with other loci, classified as intergenic lncRNAs (lncRNAs); (2) overlap with sense intron; (3) overlap with antisense intron; (4) overlap with sense exon; and (5) overlap with antisense exon. The last four classes include two conditions: (a) the lncRNA contains the intron or exon; (b) the lncRNA is contained within the intron or exon.

### 5.8. Expression Analysis

The FPKM value was converted to a read count value by Stringtie (v1.3.3) software. The differentially expressed protein-coding genes and lncRNA were identified by DESeq2 software [72], and differentially expressed protein-coding genes and lncRNAs were filtered according to *p* < 0.05 after Benjamini-adjusted and |log2 (fold change)| >1. The edgeR software identifies differentially expressed miRNAs. and miRNAs with |log2 (FC)|>1 & FDR < 0.05 were considered to be differentially expressed miRNAs.

### 5.9. Functional Enrichment Analyses for miRNA Target Genes and Protein Encoding Genes

Differential expression of miRNA target genes was predicted by TargetScan and miRBase databases. DAVID (http://david.abcc.ncifcrf.gov/) was used for GO and KEGG pathway analyses of differentially expressed mRNA and miRNA target genes. Kyoto Encyclopedia of Genes and Genomes (KEGG) pathways also were visualized by KOBAS v3.0. The pathways with *p* value < 0.05 were defined as significant threshold.

### 5.10. miRNA-mRNA Targeted Association Analysis

The target gene is matched with the differentially expressed protein-coding gene, and the miRNA and protein-encoding gene with a targeted regulatory relationship were retained. Using |log2 (FC)|>1 as a threshold, a larger likelihood of miRNA and DEGs was screened for subsequent analysis.

### 5.11. Construction of the lncRNA mRNA Co-expression Network

We constructed the lncRNA-mRNA co-regulated network using differentially expressed lncRNAs and mRNAs. Search for all differential protein encoded genes in the 100kb upstream and downstream of differentially expressed lncRNA, and use R to calculate their Pearson correlation coefficients (PCCs). The PCC value > 0.80 and *p* < 0.05 were considered significant pair. The lncRNA-mRNA co-expression network diagrams were visualized on Cytoscape v3.6.1 software [73]. 

### 5.12. Quantitative Real-time Polymerase Chain Reaction (qRT-PCR) Analysis

Total RNA was isolated from liver tissue (50mg) using TRIzol (Takara, Shanghai, China). The synthesis of single-stranded cDNA from 5 µg of RNA was performed using EasyScript One-Step gDNA Removal and cDNA Synthesis SuperMix (Solarbio, Beijing, China). The mRNA was reverse transcribed into cDNA under a reaction system of 20 uL and incubated at 42 °C for 15 min. The cDNA was used as a template for qRT-PCR analysis by TransStart To Green qRT-PCR SuperMix (Solarbio, Beijing, China). qRT-PCR amplification was performed in a 10 μL volumes with 100–150 ng of cDNA. Follow the instructions in the manual under the following conditions: pre-denatured at 94 °C for 30 s, then 40 cycles of 94° C for 5 s, 55° C for 15 s, and 72° C for 10 s, followed by a final extension at 72° C for 7 min. qRT-PCR products were verified on 1.5% agarose gels. The primer sequences were listed in Table 4. Data analysis was carried out using the comparative 2^−ΔΔCT^ method [74].

## Figures and Tables

**Table 1:** Liver lncRNA and mRNA sequencing data description and comparison rate statistics.

Category	Sample Name	Raw Reads (Mb)	Clean Reads (Mb)	Effective Rate (%)	Raw Base (Gb)	Clean Base (Gb)	Mapping Rate (%)
lncRNA	CB-1	99.97	97.62	97.64	15.00	14.64	96.20
	CB-2	116.55	114.09	97.89	17.49	17.11	95.80
	CB-3	89.33	87.35	97.78	13.39	13.10	95.70
	TB3-1	52.69	52.23	99.13	15.81	15.67	92.10
	TB3-2	57.12	56.64	99.17	17.14	16.99	92.70
	TB3-3	53.94	53.53	99.25	16.18	16.06	92.80
miRNA	CB-1	12.58	10.96	87.15	0.63	0.24	76.00
	CB-2	12.18	10.75	88.28	0.61	0.24	74.20
	CB-3	11.87	11.87	87.56	0.59	0.23	76.70
	TB3-1	11.09	9.53	85.93	0.53	0.48	86.60
	TB3-2	10.08	8.24	81.75	0.49	0.54	81.60
	TB3-3	12.69	10.71	84.39	0.61	0.41	82.20

Note: TB is treatment group chickens fed with 1.2 mg/kg AFB1, CB is control group of chickens fed with 0.0 mg/kg AFB1.

**Table 2:** miRNA–mRNA targeting association in the liver.

Gene Symbol	Log (FC)	miRNA	Log (FC)	Gene Symbol	Log (FC)	miRNA	Log (FC)
*ARHGEF7*	−1.10	gga-miR-219b-3p	6.04	*ENPP2*	−1.28	gga-miR-140-5p	−3.69
*BCL6*	−2.40	gga-miR-190a-3p	5.44	*JAG1*	−1.62	gga-miR-19b-5p	−5.22
*CLOCK*	−1.22	gga-miR-142-5p	6.54	*JAG1*	−1.62	gga-miR-34a-5p	−5.95
*CLOCK*	−1.22	gga-miR-301b-3p	3.87	*JAG1*	−1.62	gga-miR-34a-5p	−5.95
*CLOCK*	−1.22	gga-miR-301a-3p	2.37	*MEF2C*	−1.36	gga-miR-107-5p	−4.00
*DDX6*	−1.23	gga-miR-301b-3p	3.87	*PRKCE*	−1.07	gga-miR-32-5p	−6.13
*DDX6*	−1.23	gga-miR-301a-3p	2.37	*SGK1*	−1.14	gga-miR-365-3p	−4.59
*DUSP8*	−1.45	gga-miR-190a-3p	5.44	*TAB2*	−1.17	gga-miR-140-5p	−3.69
*MYH10*	−1.45	gga-miR-142-3p	5.91	*BTG1*	4.33	gga-miR-301b-3p	3.87
*MYH9*	−1.26	gga-miR-133b-3p	4.65	*BTG1*	4.33	gga-miR-138-5p	3.30
*MYH9*	−1.26	gga-miR-133c-3p	2.60	*BTG1*	4.33	gga-miR-301a-3p	2.37
*NFATC2*	−1.26	gga-miR-301b-3p	3.87	*CPEB1*	2.97	gga-miR-301b-3p	3.87
*NFATC2*	−1.26	gga-miR-301a-3p	2.37	*CPEB1*	2.97	gga-miR-301a-3p	2.37
*SGK1*	−1.14	gga-miR-142-5p	6.54	*DERA*	1.39	gga-miR-1454-3p	5.58
*SGK1*	−1.14	gga-miR-1454-3p	5.58	*LYPLA1*	1.04	gga-miR-138-5p	3.30
*TAB2*	−1.17	gga-miR-219b-3p	6.04	*PPARG*	1.15	gga-miR-301b-3p	3.87
*TAB2*	−1.17	gga-miR-142-3p	5.91	*PPARG*	1.15	gga-miR-301a-3p	2.37
*TFDP2*	−1.01	gga-miR-107-3p	6.31	*PRKG1*	3.44	gga-miR-107-3p	6.31
*ULK2*	−1.63	gga-miR-301b-3p	3.87	*RBPJ*	1.86	gga-miR-219b-3p	6.04
*ULK2*	−1.63	gga-miR-301a-3p	2.37	*RHEB*	2.12	gga-miR-142-3p	5.91
*AP3S1*	1.44	gga-miR-32-3p	−4.69	*SAT1*	1.57	gga-miR-219b-3p	6.04
*RHEB*	2.12	gga-miR-107-5p	−4.00	*WEE1*	1.46	gga-miR-1454-3p	5.58
*B3GALT2*	−1.32	gga-miR-107-5p	−4.00	*YWHAZ*	1.29	gga-miR-193a-3p	2.95
*B3GALT2*	−1.32	gga-miR-32-5p	−6.13				

Note: LogFC means abbreviation of log2 (fold change). Log (FC)> 1 represents miRNA or gene with upregulated expression, log (FC) <1 represents miRNA or gene with downregulated expression.

**Table 3:** Candidate lncRNA and protein-coding genes involved in fat metabolism and apoptosis.

Lnc_id	Chr	Start	End	Gene Transcription ID	Gene Name	Start	End	PCC	*p*-Value
TU10057	12	4774308	4829222	ENSGALT00000007976	*PPARG*	4859854	4880521	0.87	0.025
TU37186	5	16757648	16759893	ENSGALT00000011621	*FADS2*	16777263	16794363	0.88	0.019
TU33274	4	13925955	13927633	ENSGALT00000013126	*ACSL4*	13835063	13847676	−0.81	0.049
TU35007	4	60355237	60362380	ENSGALT00000077492	*ADH6*	60350736	60361473	0.96	0.002
TU35007	4	60355237	60362380	ENSGALT00000052318	*ADH6*	60363449	60368454	0.96	0.002
TU35007	4	60355237	60362380	ENSGALT00000037633	*ADH6*	60350928	60355023	0.96	0.002
TU35007	4	60355237	60362380	ENSGALT00000032123	*ADH6*	60350738	60361446	0.96	0.002
TU35007	4	60355237	60362380	ENSGALT00000032121	*ADH6*	60356945	60368587	0.96	0.002
TU26609	26	4070789	4074525	ENSGALT00000004076	*PPARD*	4089638	4106262	−0.87	0.023
TU3310	1	78318443	78322786	ENSGALT00000023745	*ATG8*	78391093	78400942	0.89	0.019
TU45776	9	14829770	14830278	ENSGALT00000011898	*BCL6*	14830574	14846807	−0.83	0.043
TU1868	1	50879489	50880054	ENSGALT00000084251	*CSNK1E*	50966184	50982191	−0.85	0.032
TU1868	1	50879489	50880054	ENSGALT00000053457	*CSNK1E*	50962355	50982806	−0.85	0.032

**Table 4:** The primer sequences used in this study.

Transcript Name	Forward	Reverse
DTU45776	CCTAAGACCGAAGCCAGCATC	AAGCGAATCCCACGAGCAG
DTU10057	GGCAGCAAGGCATCAGAAGG	TGCAGAGTCGAAACAACCCATG
gga-miR-301b-3p	GCAGTGCAATAGTATTGTCAAAGCA	TGCTTTGACAATACTATTGCACTGC
gga-miR-190a-3p	CCGCCCTATATATCAAACATATTCC	GGAATATGTTTGATATATAGGGCGG
β- actin	GTCCACCGCAAATGCTTCTA	AGCCATGCCAACTCCGTCTT
U6	GGAACGATACAGAGAAGATTAGC	TGGAACGCTTCACGAATTTGCG
*SGK1*	AATGGCACAACCTCCACCTTC	AAGACTGCTCCAAGGCACCAC
*PPARG*	GACCAAAGCCAAGGCAAGG	TTGATCTGATCTTCTCCCATCCTTA
*RHEB*	ACGCACTCAGCACAAAGGACC	TTCCACCACCTTGCCCTGA
*ATG8*	CCTGGCTCGGCTCCCTTCTC	ATCTTTGCCGACTCGACACATCTG
*BCL-6*	CGAGGAGCCCCCGAGCAAATCAGG	CCGCGGTGCTCAGGAGAAGGGAACT

## Supplementary Materials

The following are available online at https://www.mdpi.com/2072-6651/12/5/290/s1, Table S1. The expression profiles of identified miRNA (FPKM > 0) lncRNA and protein-coding genes (FPKM > 0). (XLSX), Table S2. Differentially expressed mRNAs, lncRNAs, miRNAs. (XLSX), Table S3. The information of 164 lncRNAs correspond to 186 mRNAs. in the 100kb range. (XLSX), Table S4. Enrichment Analysis of KEGG Signaling Pathway of 29 mRNAs. (XLSX), Table S5. Enrichment Analysis of KEGG Signaling Pathway of 186 mRNAs. (XLSX).
